# Magnified Neural Envelope Coding Predicts Deficits in Speech Perception in Noise

**DOI:** 10.1523/JNEUROSCI.2722-16.2017

**Published:** 2017-08-09

**Authors:** Rebecca E. Millman, Sven L. Mattys, André D. Gouws, Garreth Prendergast

**Affiliations:** ^1^Manchester Centre for Audiology and Deafness, Division of Human Communication, Deafness and Hearing, School of Health Sciences, Faculty of Biology, Medicine and Health, University of Manchester, Manchester M13 9PL, United Kingdom,; ^2^Department of Psychology, University of York, York YO10 5DD, United Kingdom, and; ^3^York Neuroimaging Centre, The Biocentre, York Science Park, Heslington, York YO10 5NY, United Kingdom

**Keywords:** envelope coding, magnetoencephalography, sensorineural hearing loss, speech perception

## Abstract

Verbal communication in noisy backgrounds is challenging. Understanding speech in background noise that fluctuates in intensity over time is particularly difficult for hearing-impaired listeners with a sensorineural hearing loss (SNHL). The reduction in fast-acting cochlear compression associated with SNHL exaggerates the perceived fluctuations in intensity in amplitude-modulated sounds. SNHL-induced changes in the coding of amplitude-modulated sounds may have a detrimental effect on the ability of SNHL listeners to understand speech in the presence of modulated background noise. To date, direct evidence for a link between magnified envelope coding and deficits in speech identification in modulated noise has been absent. Here, magnetoencephalography was used to quantify the effects of SNHL on phase locking to the temporal envelope of modulated noise (envelope coding) in human auditory cortex. Our results show that SNHL enhances the amplitude of envelope coding in posteromedial auditory cortex, whereas it enhances the fidelity of envelope coding in posteromedial and posterolateral auditory cortex. This dissociation was more evident in the right hemisphere, demonstrating functional lateralization in enhanced envelope coding in SNHL listeners. However, enhanced envelope coding was not perceptually beneficial. Our results also show that both hearing thresholds and, to a lesser extent, magnified cortical envelope coding in left posteromedial auditory cortex predict speech identification in modulated background noise. We propose a framework in which magnified envelope coding in posteromedial auditory cortex disrupts the segregation of speech from background noise, leading to deficits in speech perception in modulated background noise.

**SIGNIFICANCE STATEMENT** People with hearing loss struggle to follow conversations in noisy environments. Background noise that fluctuates in intensity over time poses a particular challenge. Using magnetoencephalography, we demonstrate anatomically distinct cortical representations of modulated noise in normal-hearing and hearing-impaired listeners. This work provides the first link among hearing thresholds, the amplitude of cortical representations of modulated sounds, and the ability to understand speech in modulated background noise. In light of previous work, we propose that magnified cortical representations of modulated sounds disrupt the separation of speech from modulated background noise in auditory cortex.

## Introduction

Hearing loss is a major health issue that affects >40% of the population who are 60 years of age or older (Agrawal et al., 2008). The most common form of hearing loss, sensorineural hearing loss (SNHL), is associated with damage to the hair cells in the cochlea. In addition to elevating audiometric thresholds, SNHL alters the perception and neural representations of sounds. For example, the reduction in fast-acting compression associated with outer hair cell dysfunction exaggerates the perceived fluctuations in the amplitude of modulated sounds (Moore et al., 1996). However, magnified neural coding of the temporal envelope of modulated sounds may not be beneficial for real-world listening situations. Simulations of SNHL in normal-hearing listeners suggest that magnified neural coding of sound envelopes has a detrimental effect on the ability to understand speech in the presence of modulated background noise (Moore and Glasberg, 1993). Magnified envelope coding may distract hearing-impaired listeners from using other auditory cues to aid speech perception in noise (Kale and Heinz, 2010; Henry et al., 2014; Zhong et al., 2014).

Direct evidence for a link between magnified envelope coding and deficits in the ability to understand speech in modulated noise backgrounds is lacking. To address this issue, we used magnetoencephalography (MEG) to measure cortical phase-locking to the temporal envelope of modulated noise (envelope coding) in normal-hearing (NH) and hearing-impaired listeners with bilateral SNHL. We considered both groups because NH and SNHL listeners are known to differ in their ability to “listen in the dips” of modulated noise to aid speech perception; that is, to take advantage of the higher signal-to-noise ratio (SNR) during amplitude minima in modulated background noise (Duquesnoy, 1983; Festen and Plomp, 1990; Moore et al., 1999; Moore, 2008).

We tested the hypothesis that magnified cortical envelope coding is associated with deficits in speech perception in modulated noise backgrounds and, in addition, we aimed to quantify the effects of SNHL on the fidelity of cortical envelope coding. Indeed, speech perception in modulated noise may partially rely on accurate coding of the amplitude minima in the envelope shape of a fluctuating background noise (Grose et al., 2009). The fidelity of envelope coding in human auditory cortex may provide a measure of temporal processing that is directly related to the ability to benefit from the temporal dips in modulated maskers. The amplitude of the phase-locked response to the modulated noise was measured using a general linear model (GLM) approach. Cross-correlation analyses provided a measure of the fidelity of envelope coding, that is, a measure of the accuracy with which the temporal structure of the predictor is represented by the measured cortical activity (see Materials and Methods: “MEG analysis”).

In the present study, we found that both the amplitude and the fidelity of cortical envelope coding of square-wave-modulated noise were enhanced in SNHL listeners compared with NH listeners. Cortical envelope coding of modulated noise was lateralized toward right auditory cortex within both listeners groups, consistent with asymmetric sampling in time (AST) theory (Poeppel, 2003). AST theory predicts that the cortical coding of slowly fluctuating sounds (∼4 Hz) is lateralized toward the right hemisphere because neurons in right auditory cortex integrate preferentially over time windows of ∼250 ms. Enhanced envelope coding was more evident in right auditory cortex despite the symmetrical hearing loss of the SNHL listeners. These results permit new insights into the functional lateralization of enhanced cortical envelope coding in SNHL listeners. Critically, both hearing thresholds and, to a lesser extent, the amplitude of envelope coding in left posteromedial auditory cortex were predictive of deficits in the identification of speech sentences presented against a background of modulated noise.

## Materials and Methods

### 

#### Participants

Seventeen (4 male) NH listeners (mean age = 57 years, SD = 5 years) and 17 (8 male) SNHL listeners (mean age = 61 years, SD = 11 years), all right-handed native English speakers, participated in the study. A one-way ANOVA showed that there was no significant age difference between the NH and SNHL listeners (*F*_(1,32)_ = 1.42, *p* = 0.24). All listeners provided written informed consent in accordance with the Declaration of Helsinki and were paid for their participation in the study. The study was approved by the Research Governance Committee at the University of York.

#### Audiological assessment

Audiometric thresholds were measured in accordance with the procedures recommended by the British Society of Audiology (British Society of Audiology, 2004). Pure tone air conduction thresholds were measured for all listeners and bone conduction thresholds were also measured for hearing-impaired listeners. Audiometric thresholds were measured for frequencies of 0.5, 1, 2, and 4 kHz only because stimuli were band-pass filtered with cutoff frequencies of 0.5 and 4 kHz (linear-phase FIR digital filter followed by sixth-order Butterworth filter). The NH listeners had clinically normal hearing in both ears, defined here as pure tone audiometric thresholds of no more than 20 dB HL for octave frequencies between 0.5 and 4 kHz. All SNHL listeners had a bilateral mild to moderate hearing loss, defined as audiometric thresholds >20 dB HL in both ears for at least one test frequency.

Hearing thresholds for NH and SNHL listeners were analyzed in a 2 (hearing status) × 2 (ear) × 4 (hearing test frequency) mixed repeated-measures ANOVA. Greenhouse–Geisser corrected *p*-values are reported where necessary.

#### Behavioral measures of speech perception in noise

Listeners were seated in a double-walled sound-attenuating booth. Stimuli were delivered diotically through Sennheiser HD 650 headphones. Stimuli were played through an E-MU soundcard using custom MATLAB (The MathWorks) routines. Linear frequency-dependent amplification (Moore and Glasberg, 1998) was used to increase the audibility of the stimuli for SNHL listeners (see “Audibility of auditory stimuli” section).

The ability to benefit from the temporal dips in modulated maskers was measured using target sentences from the IEEE corpus (Rothauser et al., 1969). The entire list of IEEE sentences was sorted based on their duration: 120 sentences of the IEEE shortest sentences (mean duration = 2.22 s, SD = 0.06 s) were used for the behavioral testing and a further 30 sentences were used for training before behavioral testing. Sentences contained four or five key words (mean number of key words = 4.92, SD = 0.27); for example, “The lake sparkled in the red hot sun” (keywords underlined). At the end of each sentence, listeners were instructed to type any words that they could understand using a computer keyboard.

Keyword identification was measured in a masking noise that was spectrally matched to the long-term power spectrum of the speech sentences. The masker was either unmodulated (see Fig. 3*A*) or 100% modulated with a 2 Hz square wave (50% duty cycle) (see Fig. 3*B*). The “on” and “off” periods of the modulated masker were ∼250 ms in duration because the on/off slopes of the square-wave modulator were shaped with 5 ms cosine squared ramps. The most common rise time in the temporal envelopes of IEEE speech sentences is 12 ms (79 modulations/s) across auditory 128 filters, but many envelope fluctuations with a rise time of 4–6 ms (61 modulations/s) are also present (Prendergast et al., 2011).

Different noise samples were selected for each presentation. When the noise was modulated, the first period of the noise was always “on” and therefore the first 250 ms portion of each sentence was masked. Therefore the modulated noise was always in phase with the sentence onset, which would make the amplitude minima in the modulated noise predictable given that only one modulation rate was tested. The predictability of the temporal dips in the modulated masker should improve absolute performance in the modulated masker condition and increase the amount of speech MR relative to a modulated masker with a random starting phase. The speech-in-noise stimuli were ramped on and off using a 25 ms raised cosine function. SNRs were fixed at −4, −8, and −12 dB. Twenty sentences were randomly assigned to each of three SNR conditions (−4, −8, and −12 dB) and two masker conditions (unmodulated, modulated). Because the lower SNRs (−8 and −12 dB) resulted in floor performance in the unmodulated masker condition, speech MR was only measured for −4 dB SNR.

Speech masking release, the ability to benefit from the temporal dips in modulated maskers, was defined as the difference in performance (percentage of sentence keywords correctly identified) between the unmodulated and modulated maskers presented at a fixed SNR of −4 dB (Bernstein and Grant, 2009; Gregan et al., 2013). Individual scores for correct identification of keywords were transformed into rationalized arcsine units (Studebaker, 1985) for statistical analyses.

The percentage of sentence keywords correctly identified were analyzed in a 2 (hearing status) × 2 (masker type) mixed repeated-measures ANOVA. The number of correctly identified keywords in each keyword position within a sentence was analyzed in a 2 (hearing status) × 5 (keyword position) mixed repeated-measures ANOVA. Greenhouse–Geisser corrected *p*-values are reported where necessary.

#### Audibility of auditory stimuli

The Cambridge formula (Moore and Glasberg, 1998) was used to improve audibility for the SNHL listeners. Based on the audiometric threshold of each listener, gains specified at audiometric frequencies between 0.5–4 kHz were prescribed by the Cambridge formula (CAMEQ):

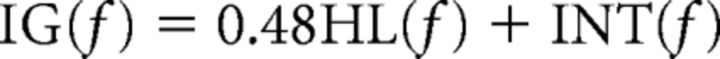
 where IG(*f*) is the insertion gain in dB at frequency *f*, HL(*f*) is the hearing loss in dB at frequency *f*, and INT(*f*) is a frequency-dependent intercept. The CAMEQ was applied after speech and noise were mixed at −4 dB SNR. The prescribed gains were applied to the processed sounds using a linear-phase FIR digital filter (Vickers et al., 2001; Hopkins, 2009); that is, the MATLAB FIR2 function with 443 taps (Hopkins, 2009). For SNHL listeners, the CAMEQ was used to apply frequency-dependent amplification based on individual audiometric thresholds (0.5–4 kHz), calculated, and applied separately for each ear before stimulus presentation. The stimuli for NH listeners were also subjected to the signal processing pipeline required to apply the CAMEQ, but the frequency-dependent amplification was not applied. The stimulus level was 65 dB SPL for NH listeners.

#### MEG procedure

Listeners who had taken part in the behavioral experiment were invited to take part in a separate MEG session at a later date. The modulated noise, which was used to measure the perceptual benefit of the modulated masker for speech perception in noise, was also used to measure cortical envelope coding. One hundred different samples of modulated noise were played to the listeners, with one sample of modulated noise presented per trial. The duration of each sample of modulated noise was 2000 ms. One hundred epochs of silence, 100 epochs of spoken sentences mixed with unmodulated noise (SNR = −4 dB), and 100 epochs of spoken sentences mixed with modulated noise (SNR = −4 dB) were also presented during the MEG recording. The speech sentences used in the MEG session were from the IEEE corpus (Rothauser et al., 1969), but listeners had not been exposed to these sentences before the MEG recording. The duration of each epoch was increased to 3000 ms through the addition of silence to the end of each stimulus. Stimuli were presented to both ears through ER30 insert earphones (Etymotic Research). Linear-frequency-dependent amplification was applied to stimuli for SNHL listeners (see “Audibility of auditory stimuli” section). Before linear amplification, the stimulus level was 65 dB SPL.

Data were collected using a Magnes 3600 whole-head 248-channel magnetometer (originally manufactured by 4-D Neuroimaging). The data were recorded at a sample rate of 678.17 Hz and digitally filtered between 1 and 200 Hz online. Participants were asked to close their eyes during the MEG recording. Catch trials (10% of the total number of trials) were used to maintain a constant level of alertness. During a catch trial, participants were presented with an auditory cue (the word “rate”), which required a button press on a response box. The auditory cue prompted participants to indicate, via the button press, whether the previous trial contained an intelligible (masked) speech sentence, an unintelligible (masked) speech sentence, or a sound containing no speech sentence, that is, modulated noise alone.

MEG data were coregistered with the anatomical magnetic resonance (MR) scans of individual listeners. Before MEG data acquisition, individual facial and scalp landmarks were spatially coregistered using a Polhemus Fastrak System. The landmark locations in relation to the sensor positions were derived on the basis of a precise localization signal provided by five spatially distributed head coils with a fixed spatial relation to the landmarks. These head coils provided a measurement of the listeners' head movement at the beginning and end of each recording. The landmark locations were matched with the individual listeners' anatomical MR scans using a surface-matching technique adapted from Kozinska et al. (2001). T1-weighted MR images were acquired with a 3.0 T Signa Excite HDx system (General Electric) using an 8-channel head coil and a 3-D Fast Spoiled Gradient Recall Sequence (TR/TE/flip angle = 8.03 ms/3.07 ms/20°, spatial resolution of 1.13 mm × 1.13 mm × 1.0 mm, with an in-plane acquisition matrix of 256 × 256 and >176 contiguous slices).

#### MEG analysis

The raw data from each epoch were inspected visually. Epochs containing physiological or nonphysiological artifacts were removed.

##### Derivation of spatial filters.

**Figure 1.**
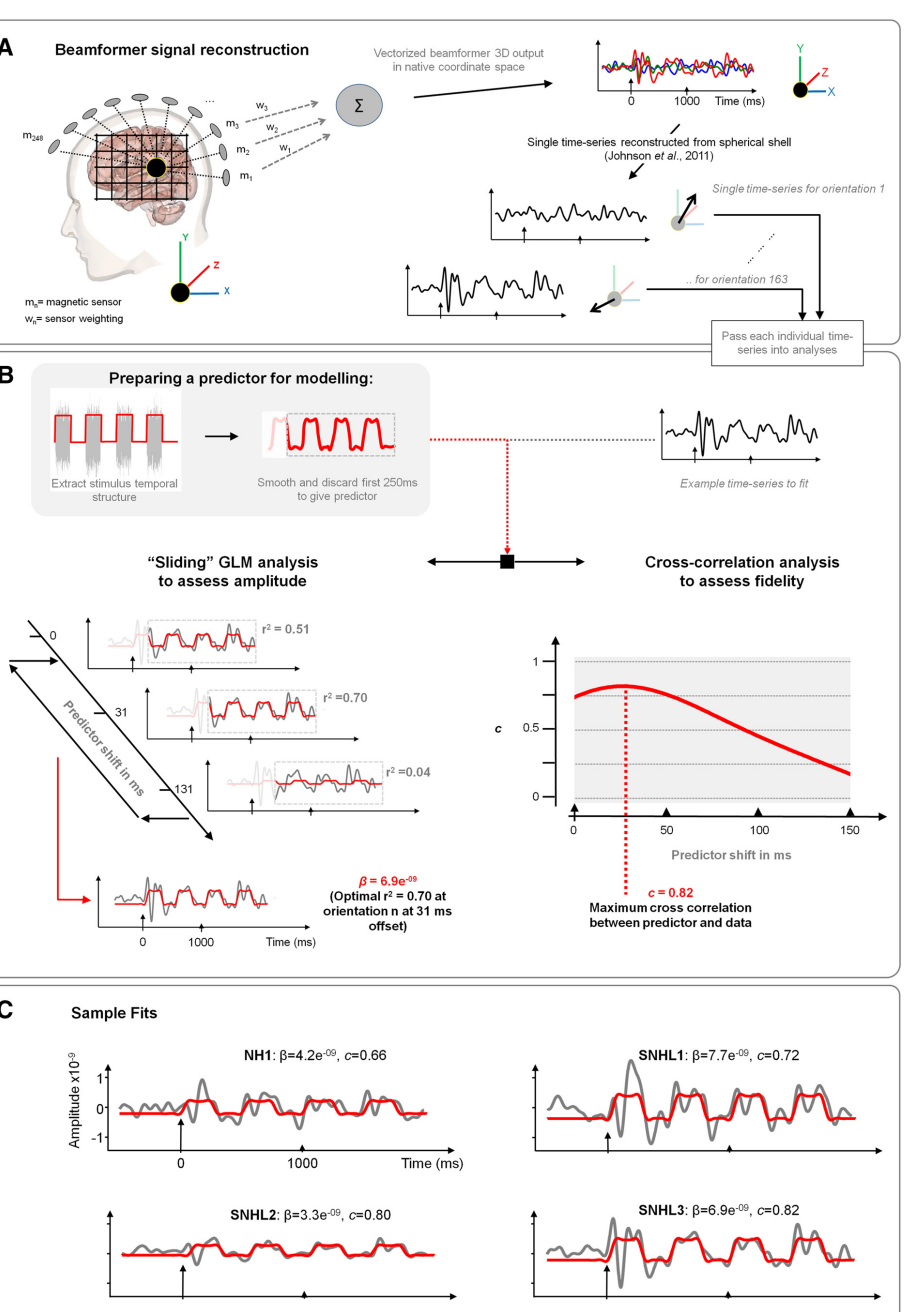
MEG analysis. ***A***, MEG data were analyzed using a vectorized, linearly constrained minimum variance beamformer and single time series were reconstructed in multiple orientations. ***B***, Temporal structure of the modulated noise was used to generate a predictor for the MEG analyses. For the analyses of the amplitude of the response, the orientation of the spatial filters was optimized on the basis of the direction that gave the best *r*^2^ of the GLM fit. The lag between the MEG response and the GLM predictor was also optimized on an individual basis to yield the best *r*^2^ of the GLM fit. For the analyses of the fidelity of the response, the orientation of the spatial filters and the stimulus–response lag was optimized based on the maximum cross-correlation coefficient. ***C***, Examples of GLM analyses of cortical envelope coding in right HG in a NH listener and SNHL listeners. For the NH listener (NH1), both the amplitude (β = 4.2e-9) and fidelity (*c* = 0.66) of envelope coding are moderate. For SNHL1, the amplitude of envelope coding is large (β = 7.7e-9), but the fidelity of envelope coding is relatively modest (*c* = 0.72). For SNHL2, the amplitude of the response is modest (β = 3.3e-9), but the fidelity is high (*c* = 0.80). For SNHL3, the amplitude of the response is large (β = 6.9e-9) and the fidelity of the response is high (*c* = 0.82).

In this study, a vectorized, linearly constrained minimum variance beamformer (Van Veen et al., 1997; Huang et al., 2004) was used to obtain the spatial filters with a multiple-spheres head model (Huang et al., 1999). The beamformer grid size was 5 mm. The three orthogonal spatial filters were implemented as a single 3-D system (Johnson et al., 2011) (Fig. 1*A*). Spatial filters were generated with a time window of 2500 ms, including 500 ms before stimulus presentation, and a 1–10 Hz band-pass filter.

##### Analysis of spatial filter outputs.

The amplitudes of the MEG responses were analyzed using a GLM approach as follows:

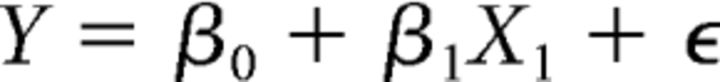
 where each observed data point (*Y*) is the sum of a constant term (β_0_), the β of interest (β_1_), the stimulus (*X*_1_), and residual error (ε). GLM analyses were performed using the *regstats* function in MATLAB (The MathWorks). The outputs of the beamformer spatial filters were the observed data. The temporal structure of the square-wave-gated noise was the predictor after the predictor was smoothed (Brookes et al., 2004) using a low-pass filter with a cutoff frequency of 10 Hz (Fig. 1*B*). The β_1_ of the GLM gives the amplitude of envelope coding. The *r*^2^ of the GLM was used to optimize the orientation of the spatial filters (Millman et al., 2015) (Fig. 1*B*). The *r*^2^ of the model fit, rather than β_1_, was chosen as the optimizing metric to increase the likelihood that the spatial filter output resembled a square wave. Table 1 shows the mean GLM *r*^2^ for both listener groups in each cortical area of interest. The overall GLM fit was significant in all individual listeners (*p* < 0.001).

**Table 1.** GLM goodness-of-fit (*r*^2^) was used to optimize the GLM analyses of amplitude of the response to square-wave modulated noise (see Fig. 1*B*)

	GLM *r*^2^			
	Right HG	Left HG	Right STG	Left STG
NH	0.46 (0.14)	0.34 (0.14)	0.42 (0.13)	0.37 (0.17)
SNHL	0.56 (0.14)	0.35 (0.13)	0.59 (0.10)	0.4 (0.12)

Means and SD (in parentheses) of the GLM *r*^2^ are shown for NH and SNHL listeners in each cortical LOI.

The fidelity of envelope coding was measured using cross-correlation in the time domain using the MATLAB *xcov* function (Abrams et al., 2008; Nourski et al., 2009; Millman et al., 2013). Cross-correlation analyses were performed between the smoothed square-wave predictor and the phase-locked MEG responses. Cross-correlation coefficients (*c*) were Fisher transformed before statistical analyses (Abrams et al., 2008; Millman et al., 2013, 2015).

Measures of cortical envelope coding were restricted to the “envelope following period” (Abrams et al., 2008) from 250–2000 ms after stimulus presentation. A variable lag of 0–150 ms (Nourski et al., 2009; Millman et al., 2013) was introduced between the onset of stimulus presentation and the MEG signal to identify the lag that resulted in the best *r*^2^ of the GLM fit or maximum *c* for cross-correlation analyses (Fig. 1*B*). These lags were optimized for individual participants.

The MEG data were analyzed in a 2 (hearing status) × 2 (hemisphere) × 2 (location) repeated-measures ANOVA. The optimal stimulus–response lags were analyzed in a 2 (hearing status) × 2 (analysis method) × 2 (hemisphere) × 2 (location) repeated-measures ANOVA.

##### Locations of spatial filters.

The MEG data were analyzed using a location of interest (LOI) approach (Millman et al., 2013, 2015). The LOIs were left and right posteromedial Heschl's Gyrus (HG) and the posterolateral portion of left and right superior temporal gyrus (STG) (see Fig. 4*B*). These LOIs were chosen because they are known to phase lock to temporal modulations in sounds (Nourski et al., 2009, 2013; Ding and Simon, 2012; Millman et al., 2015). Posterolateral STG has been identified as playing a significant role in the perception of speech in noise: This brain region may be used when increased effort is required to understand speech in noise (Wong et al., 2009), during listening in the dips of modulated maskers (Scott et al., 2009), and for object-based neural representations (Ding and Simon, 2012).

LOIs were seeded manually in HG (Millman et al., 2013, 2015) because of the interlistener variability in the anatomy of HG (Rademacher et al., 2001). LOIs in STG were based on the region identified in left posterolateral STG (MNI −64 −30 8) by Wong et al. (2009) and its right hemisphere homolog (MNI 64 −30 8). These MNI coordinates for the LOI in posterolateral STG fall within the Harvard–Oxford probabilistic cortical atlas for Brodmann's area 42. MNI coordinates for left and right STG were transformed back into individuals' anatomical space and spatial filters were generated from these locations.

LOIs in left and right auditory cortices were selected to test the predictions of the AST model regarding right hemisphere lateralization for low-frequency envelope coding (Poeppel, 2003). LOIs in both early (HG) and associative (STG) auditory cortex were analyzed because: (1) the locus of functional asymmetry predicted by AST (Poeppel, 2003) remains unclear and (2) there is evidence that speech is at least partially separated from background noise in HG, whereas complete segregation of speech and noise occurs in posterior STG (Ding and Simon, 2012; Simon, 2015).

#### Relationship between speech perception in noise and envelope coding metrics

Hierarchical multiple regression analyses were used to assess the predictive value of the pure-tone average (PTA) hearing thresholds, age, and the envelope coding metrics (amplitude and fidelity) on speech identification in the presence of the modulated masker. Both NH and SNHL listeners were entered into the regression analysis. Known predictors of speech perception in noise, namely age (Dubno et al., 2002) and PTA (Bacon et al., 1998), were entered in the first step of the model. The envelope coding metrics were included in a stepwise fashion in the second step of the model because the relative contributions of the amplitude and fidelity of cortical envelope coding to speech perception in noise are unknown. Collinearity diagnostics did not suggest that the regression model was influenced by multicollinearity: The mean variance inflation factor (VIF) was not substantially >1 (mean VIF = 1.25, SD = 0.44) and the maximum VIF was <10 (maximum VIF = 2.55) (Bowerman and O'Connell, 1990).

## Results

### Symmetrical hearing thresholds in NH and SNHL listeners

**Figure 2.**
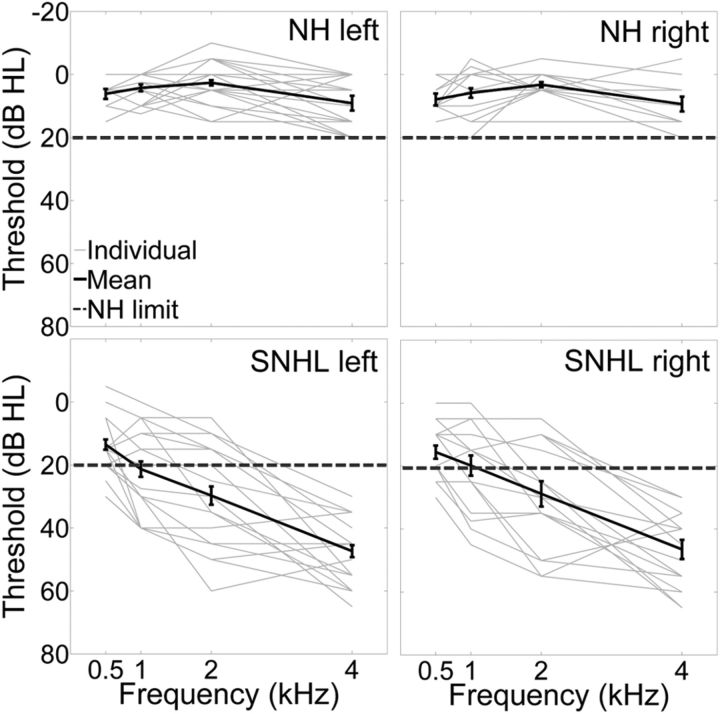
Hearing thresholds measured with pure tone air conduction audiometry for the left ear (left) and right ear (right) of the NH (top) and SNHL (bottom) listeners. The solid gray lines represent individual audiometric thresholds. The solid black lines represent mean audiometric thresholds for NH and SNHL listeners. Error bars indicate 1 ± SEM. The dashed dark gray line indicates the hearing threshold of 20 dB HL that was considered the limit of NH.

Figure 2 shows the individual and mean hearing thresholds for the NH and SNHL listeners. A mixed repeated-measures ANOVA was used to analyze the hearing thresholds. The between-participants factor was hearing status (NH or SNHL). The within-participants factors were ear (right or left) and test frequency (0.5, 1, 2, or 4 kHz). For the comparisons of test frequency, the assumption of sphericity was violated (Mauchly's test: χ^2^(5) = 35.03, *p* < 0.001); therefore, the degrees of freedom were corrected (Greenhouse–Geisser; ε = 0.72). The ANOVA revealed main effects of hearing status (*F*_(1,32)_ = 80.38, *p* < 0.001, partial η^2^ = 0.72) and test frequency (*F*_(2.15,68.68)_ = 37.32, *p* < 0.001, partial η^2^ = 0.54). There was also a significant interaction between hearing status and test frequency ((*F*_(2.15,68.68)_ = 26.8, *p* < 0.001, partial η^2^ = 0.46). Figure 2 shows that SNHL listeners had higher (i.e., worse) hearing thresholds than NH listeners, that hearing thresholds increased as a function of test frequency, and that the difference in hearing thresholds of NH and SNHL listeners was increased for higher test frequencies. Importantly, the ANOVA showed that there was no main effect of ear (*F*_(1,32)_ = 0.28, *p* = 0.60, partial η^2^ = 0.01), demonstrating that hearing thresholds were symmetrical across ears for both listener groups.

### Speech perception in noise

**Figure 3.**
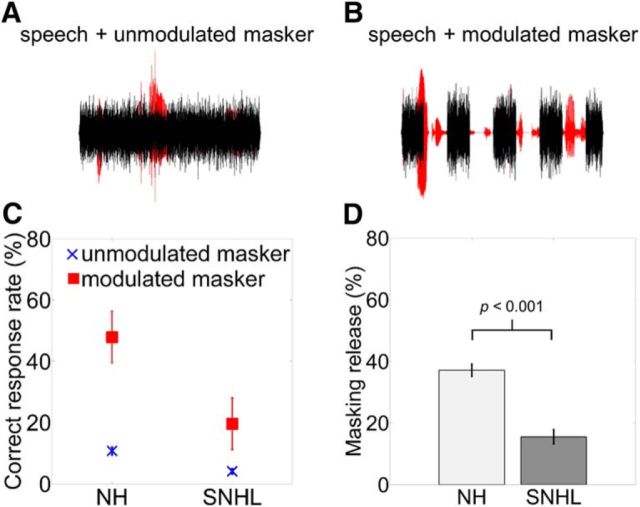
Effects of background noise on speech perception in NH and SNHL listeners. ***A***, Illustration of a spoken sentence mixed with an unmodulated noise background. ***B***, Illustration of another spoken sentence mixed with a square-wave-modulated noise background. The modulated masker provides opportunities to take advantage of the higher SNR during temporal dips in modulated background noise. ***C***, Mean accuracy (%) for NH and SNHL listeners for keyword identification of target sentences. Sentences were presented at an SNR of −4 dB against an unmodulated noise masker (crosses) and a square-wave-modulated noise masker (squares). Error bars indicate ±1 SEM. ***D***, Mean speech masking release; that is, perceptual benefit from the temporal dips in the modulated masker, for NH listeners (light gray bar) and SNHL listeners (dark gray bar) at −4 dB SNR. Error bars indicate ±1 SEM.

The percentage of correctly identified sentence keywords in the presence of unmodulated and modulated noise maskers are shown in Figure 3*C*. Speech perception in the unmodulated masker was poor for both NH and SNHL listeners. Noise modulation improved speech perception for both groups. An ANOVA was used to assess speech perception performance with the between-participants factor hearing status (NH or SNHL) and the within-participants factor masker type (unmodulated or modulated). There were significant effects of hearing status (*F*_(1,32)_ = 49.23, *p* < 0.001, partial η^2^ = 0.61) and masker type (*F*_(1,32)_ = 394.1, *p* < 0.001, partial η^2^ = 0.93) and an interaction between the two (*F*_(1,32)_ = 24.24, *p* < 0.001, partial η^2^ = 0.43). The interaction demonstrates that the NH listeners were more able to benefit more from the temporal dips in the modulated masker than SNHL listeners (Bacon et al., 1998; Gregan et al., 2013). Figure 3*D* shows the difference in speech identification in the modulated and unmodulated masker conditions for NH and SNHL listeners.

Table 2 shows the mean number of correctly identified keywords in NH and SNHL listeners as a function of the keyword position (first, second, third, fourth, or fifth) in the IEEE sentences used in the present study. To test for a predictability gain in speech comprehension for final key words (Hartwigsen et al., 2015), a mixed repeated-measures ANOVA was used to measure a change in correct keyword identification as a function of keyword position. The between-participants factor was hearing status (NH or SNHL) and the within-participants factor was keyword position (first, second, third, fourth, or fifth). For the comparisons of keyword position, the assumption of sphericity was violated (Mauchly's test: χ^2^(9) = 24.67, *p* = 0.003); therefore, the degrees of freedom were corrected (Greenhouse–Geisser; ε = 0.72). There was an effect of hearing status (*F*_(1,32)_ = 62.28, *p* < 0.001, partial η^2^ = 0.66) because NH listeners identified more keywords correctly. There was also an effect of keyword position (*F*_(2.89, 92.43)_ = 47.74, *p* < 0.001; partial η^2^ = 0.60) because the number of keywords correctly identified increased as a function of keyword position in both listener groups. There was also a significant interaction between keyword position and hearing status (*F*_(2.89, 92.43)_ = 83.67, *p* < 0.001; partial η^2^ = 0.18), suggesting that NH listeners were more able to identify the later keywords correctly.

**Table 2.** Effects of keyword position on correct keyword identification in IEEE speech sentences

	Keyword position (no. identified correctly)				
	First	Second	Third	Fourth	Fifth
NH	17.41 (6.09)	18.29 (5.10)	23.29 (6.96)	25.94 (4.99)	28.41 (4.70)
SNHL	7.24 (4.88)	6.35 (5.77)	8.17 (6.44)	10.41 (5.71)	11.94 (6.46)

Means and SD (in parentheses) of the number of keywords correctly identified in each keyword position are shown for NH and SNHL listeners.

*Post hoc* comparisons (Bonferroni corrected) were used to compare the number of correctly identified keywords in the final (fifth) position with the other keyword positions within listener groups. Consistent with a predictability gain for the final keyword (Hartwigsen et al., 2015), the number of final keywords correctly identified was significantly greater than the number of keywords in other positions in both listener groups: first versus fifth (NH *p* < 0.001, *d* = 2.83; SNHL *p* = 0.007, *d* = 0.88); second versus fifth (NH *p* < 0.001, *d* = 2.20; SNHL *p* < 0.001, *d* = 1.14); third versus fifth (NH *p* = 0.001, *d* = 0.94; SNHL *p* = 0.005, *d* = 0.80); fourth versus fifth (NH *p* = 0.007, *d* = 0.76; SNHL *p* = 0.011, *d* = 0.70).

### SNHL enhances cortical coding of modulated noise

**Figure 4.**
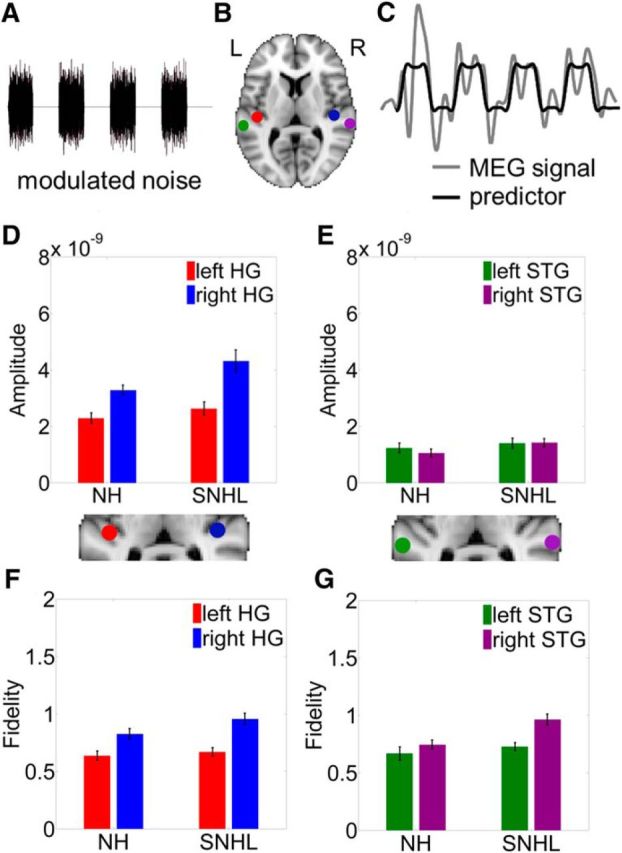
SNHL enhances cortical envelope coding of modulated noise. ***A***, Waveform of square-wave-gated modulated noise. ***B***, LOIs in left HG (red), right HG (blue), left STG (green), and right STG (purple). ***C***, Square-wave predictor (black) overlaid on the measured neural activity (gray) in a representative participant. ***D***, Mean amplitude of envelope coding in left HG (red) and right HG (blue) for NH listeners and SNHL listeners. ***E***, Mean amplitude of envelope coding in left STG (green) and right STG (purple) for NH listeners and SNHL listeners. ***F***, Mean fidelity of envelope coding in left HG (red) and right HG (blue) for NH listeners and SNHL listeners. ***G***, Mean fidelity of envelope coding in left STG (green) and right STG (purple) for NH listeners and SNHL listeners Error bars indicate ±1 SEM.

The MEG responses to the modulated noise (Fig. 4*A*) from the LOIs in HG and STG (Fig. 4*B*) were analyzed to obtain the amplitude and fidelity of cortical envelope coding (Fig. 4*C*).

The mean amplitudes of cortical envelope coding in HG (Fig. 4*D*) and STG (Fig. 4*E*) were compared in NH and SNHL listeners. Mixed repeated-measures ANOVA were used to assess the amplitudes of envelope coding in NH and SNHL listeners. The between-participants factor was hearing status (NH or SNHL). The within-participants factors were hemisphere (left or right) and location (HG or STG). A main effect of hearing status (*F*_(1,32)_ = 7.45, *p* = 0.01, partial η^2^ = 0.19) showed that envelope coding was magnified in SNHL listeners (Fig. 4*D*,*E*). Likewise, a main effect of hemisphere (*F*_(1,32)_ = 11.21, *p* = 0.002, partial η^2^ = 0.26) showed that envelope coding was lateralized toward the right hemisphere (Fig. 4*D*,*E*). There was also a main effect of location (*F*_(1,32)_ = 219.46, *p* < 0.001, partial η^2^ = 0.87) because the amplitude of envelope coding was greater in HG (Fig. 4*D*) than in STG (Fig. 4*E*). Finally, there was also a significant interaction between hemisphere and location (*F*_(1,32)_ = 33.44, *p* < 0.001, partial η^2^ = 0.51). *Post hoc* comparisons (Bonferroni corrected) revealed that this interaction between hemisphere and location was driven by significantly magnified envelope coding in right HG versus left HG (NH *p* = 0.002, *d* = 0.88; SNHL *p* = 0.002, *d* = 0.93). There was no significant hemispheric difference for STG (NH *p* = 0.52; SNHL *p* = 0.94).

The mean fidelities of cortical envelope coding are shown in HG (Fig. 4*F*) and STG (Fig. 4*G*). An ANOVA with the between-participants factor of hearing status (NH or SNHL) and the within-participants factors of hemisphere (left or right) and location (HG or STG) showed a main effect of hearing status (*F*_(1,32)_ = 8.51, *p* = 0.006, partial η^2^ = 0.21), indicating enhanced fidelity of envelope coding in SNHL listeners. There was also a main effect of hemisphere (*F*_(1,32)_ = 21.26, *p* < 0.001, partial η^2^ = 0.40): Fidelity of envelope coding was greater in the right hemisphere than the left hemisphere. A significant interaction between hemisphere and location (*F*_(1,32)_ = 5.72, *p* = 0.02, partial η^2^ = 0.15) revealed a significant difference (*post hoc* comparisons, Bonferroni corrected) in the fidelity of envelope coding in right HG versus left HG in both listener groups (NH *p* = 0.01, *d* = 0.70; SNHL *p* > 0.001, *d* = 1.11). The fidelity of envelope coding was also greater for SNHL listeners in right STG versus left STG (*p* = 0.001, *d* = 1.0) but not for NH listeners (*p* = 0.30).

The mean optimal stimulus–response lags obtained through either GLM or cross-correlation analyses are shown in Table 3. Note that the lags reported in Table 3 were calculated during the “envelope following period” from 250–2000 ms after stimulus presentation. A mixed repeated-measures ANOVA with a between-participants factor of hearing status (NH or SNHL) and the within-participants factors of analysis type (GLM or cross-correlation), hemisphere (left or right), and location (HG or STG) was performed to analyze the optimal stimulus–response lags. This ANOVA showed no effects of hearing status (*F*_(1,32)_ = 3.04, *p* = 0.09, partial η^2^ = 0.09), analysis type (*F*_(1,32)_ = 0.05, *p* = 0.82, partial η^2^ = 0.002), hemisphere (*F*_(1,32)_ = 0.35, *p* = 0.56, partial η^2^ = 0.01), or location (*F*_(1,32)_ = 2.69, *p* = 0.11, partial η^2^ = 0.08), nor any significant interactions between ANOVA terms.

**Table 3.** Optimal stimulus–response lags obtained through GLM or cross-correlation analyses

	Optimal stimulus–response lag (ms)			
	Right HG	Left HG	Right STG	Left STG
GLM				
NH	55.9 (45.0)	81.5 (51.1)	79.7 (54.1)	89.2 (54.4)
SNHL	50.4 (42.6)	58 (47.6)	71.7 (51.8)	60.2 (46.6)
Cross-correlation				
NH	55.7 (44.9)	84.8 (47.7)	82.7 (58.8)	73 (56.6)
SNHL	54.5 (44.6)	65 (52.5)	73.6 (52.4)	62.7 (51.3)

Means and SD (in parentheses) of the stimulus–response lags are shown for each LOI for NH and SNHL listeners.

### Magnified envelope coding in left HG predicts deficits in speech perception in modulated noise

**Figure 5.**
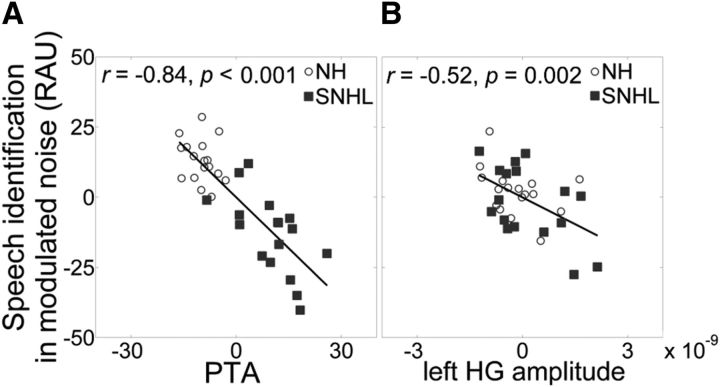
Hearing thresholds and magnified envelope coding correlate with speech identification in modulated noise. ***A***, Relationship between partial regressions for rationalized arcsine unit (RAU)-transformed speech identification in modulation noise (speech identification in modulated noise RAU) and hearing thresholds (PTA) when age is taken into account. ***B***, Relationship between partial regressions for RAU-transformed speech identification in modulation noise (speech identification in modulated noise RAU) and the amplitude of envelope coding in left HG (left HG amplitude) when both age and PTA are taken into account.

Table 4 shows the results of the hierarchical regression analyses used to assess predictors of speech perception in modulated noise. Speech identification in modulated noise was reliably predicted by the first step of the regression model (*R*^2^ = 0.65, *p* < 0.001), which included age and PTA. Consistent with previous reports, PTA contributed significantly to the model fit (*p* < 0.001) (Gregan et al., 2013), but age did not make a unique contribution (*p* = 0.46) (Füllgrabe et al., 2014). Figure 5*A* illustrates that listeners with a higher PTA, that is, greater hearing loss, did not perform as well on the speech identification task (*r* = −0.84, *p* < 0.001; Pearson's correlation coefficient, 2-tailed). The envelope coding metrics added in the second step further improved the model fit (Δ*R*^2^ = 0.09, *p* = 0.003). However, the amplitude of envelope coding in left HG was the only unique contributor to this improvement (*p* = 0.003), with magnified envelope coding associated with poor speech identification in modulated noise (*r* = −0.52, *p* = 0.002; Pearson's correlation coefficient, 2-tailed) (Fig. 5*B*).

**Table 4.** Linear model predictors (PTA, age, and envelope coding metrics) of speech perception in modulated noise

Model	Predictor	β	*p*
Step 1	Age	0.09	0.46
	PTA	**−0.84**	**<0.001**
*R*^2^ = 0.65, adjusted *R*^2^ = 0.63 for Step 1 (*p* < 0.001)			
Step 2	Age	0.12	0.25
	PTA	**−0.85**	**<0.001**
	Left HG β	**−0.31**	**0.003**
	Left HG *c*	−0.07	0.63
	Right HG β	0.03	0.81
	Right HG *c*	0.001	0.99
	Left STG β	−0.10	0.43
	Left STG *c*	0.09	0.39
	Right STG β	0.04	0.67
	Right STG *c*	0.11	0.39
Δ*R*^2^ = 0.09, adjusted *R*^2^ = 0.72 for Step 2 (*p* = 0.003)			

Model parameters include standardized beta coefficients (β) and the significance value (*p*). The *R*^2^ for the initial model step and the change in *R*^2^ (Δ*R*^2^) for the second step of the model are also reported. Values in bold indicate results at *p* < 0.05.

The significance of the link between magnified envelope coding in left HG and speech perception in noise can be explained by examining the relationships between PTA and the envelope coding metrics entered into the regression model (Table 5). Table 5 shows that scores for correct identification of keywords in modulated noise (speech) were correlated with several parameters: PTA (*r* = −0.80, *p* < 0.001), fidelity of envelope coding in right HG (*r* = −0.41, *p* = 0.02), and fidelity of envelope coding in right STG (*r* = −0.49, *p* = 0.004). Speech was also marginally correlated with the amplitude of envelope coding in both left HG (*r* = −0.31, *p* = 0.07) and right HG (*r* = −0.33, *p* = 0.06). However, the bottom row in Table 5 shows that PTA was also correlated with the same envelope coding metrics in the right hemisphere that were correlated with speech: right HG amplitude (*r* = 0.40, *p* = 0.02), right HG fidelity (*r* = 0.46, *p* = 0.006), and right STG fidelity (*r* = 0.56, *p* < 0.001). Importantly, PTA was not correlated with the amplitude of envelope coding in left HG (*r* = 0.20, *p* = 0.91).

**Table 5.** Pearson product–moment correlation coefficients for the dependent (speech) and independent variables (age, PTA, envelope coding metrics) entered into the regression model

	Speech	Age	PTA
Speech	—	−0.26	−0.80***
Age	—	—	0.41**
PTA	—	—	—

	Left HG β	Left HG *c*	Right HG β	Right HG *c*	Left STG β	Left STG *c*	Right STG β	Right STG *c*
Speech	−0.31*	−0.19	−0.33*	−0.41**	−0.20	−0.06	−0.10	−0.49***
Age	0.10	0.11	0.17	0.22	0.15	0.10	−0.19	0.21
PTA	0.20	0.009	0.40**	0.46**	−0.04	−0.008	0.15	0.56***

Correlation coefficients in boldface indicate significant results (**p ≤ 0.05; ***p ≤ 0.005). Correlation coefficients in italics are marginally significant (*p ≤ 0.07).

This robust relationship between PTA and the envelope coding metrics in the right hemisphere can account for the overall right hemisphere lateralization in the comparisons of envelope coding in NH and SNHL listeners (Fig. 4*D–G*). When the effects of PTA were controlled for in the regression analysis, envelope coding metrics in the right hemisphere were not linked with speech identification scores. However, the relationship between speech identification scores and the amplitude of envelope of coding in left HG persisted because PTA was not strongly related to the amplitude of envelope coding in left HG.

## Discussion

The present study establishes a link between the amplitude of the cortical phase-locked response to modulated sounds (envelope coding) and speech identification in the presence of modulated background noise. Both the amplitude (Wilding et al., 2012) and the fidelity (Presacco et al., 2016) of cortical envelope coding were enhanced in listeners with sensorineural hearing loss (SNHL). Our results show that SNHL exerts differential effects on envelope coding in left and right auditory cortices and posteromedial and posterolateral auditory cortices. Specifically, there was an anatomical dissociation in the effects of SNHL on envelope coding metrics in HG and STG: the amplitude of envelope coding was enhanced in HG and the fidelity of envelope coding was enhanced in HG and STG in SNHL listeners compared with NH listeners. Enhanced envelope coding was more evident in right auditory cortex in hearing-impaired listeners with a symmetrical SNHL.

### Magnified envelope coding disrupts segregation of speech and modulated noise

The results reported here link deficits in speech identification in modulated background noise with magnified cortical envelope coding. This relationship is consistent with previous work suggesting that “enhanced” envelope coding is not perceptually beneficial for speech identification in a modulated background noise (Moore and Glasberg, 1993). Heinz and colleagues proposed that magnified envelope coding distracts from other auditory cues that could be used to aid speech perception in noise (Kale and Heinz, 2010; Henry et al., 2014; Zhong et al., 2014).

Our results suggest that the envelope coding of both speech and modulated background noise are magnified in SNHL listeners. Speech identification in background noise is partly determined by interactions between the target and masker temporal envelopes (Stone et al., 2012). Magnified envelope coding of both speech and modulated background noise may impair the perception of speech envelope cues, either within the same frequency region as the modulated masker, or in a different frequency region to the modulated masker through the process of modulation discrimination interference (MDI) (Yost et al., 1989; Yost and Sheft, 1989; Bacon and Moore, 1993). MDI may partly arise from perceptual grouping of the target and masker envelopes (Yost and Sheft, 1989; Moore and Shailer, 1992), making it difficult to distinguish the target envelope from the masker envelope.

Sek et al. (2015) measured MDI in NH and SNHL listeners and found that modulation detection thresholds in hearing-impaired listeners were more susceptible to MDI when the masker modulation frequency was the same as the target modulation frequency. Sek et al. (2015) argued that SNHL-induced magnified envelope coding of highly modulated signals, such as those used in the present study, may “saturate” the sensation of fluctuations in the level of modulated sounds. This saturation in the coding of magnified envelopes could facilitate MDI between speech and modulated noise envelopes, thereby disrupting the segregation of speech cues from modulated background noise. We propose that deficits in speech identification in modulated noise in SNHL listeners could arise because magnified envelope coding interrupts the separation of speech from modulated background noise. Our results implicate left HG as an important cortical location for the segregation of speech and modulated background noise.

### Effects of SNHL on cortical envelope coding

The effects of SNHL on the magnitude of envelope coding can be linked with physiological changes at the level of the cochlea, that is, a reduction or loss of fast-acting compression that magnifies the perceived fluctuations in the amplitude of modulated sounds in comparison with an NH ear (Moore and Glasberg, 1993; Moore et al., 1996). Therefore, the magnified cortical envelope coding identified here could be inherited from the SNHL-induced changes in the auditory periphery or auditory nerve (Kale and Heinz, 2010, 2012). However, the anatomical dissociations in the effects of SNHL on cortical envelope coding metrics identified in the present study suggest some influence of SNHL on central auditory processing. Specifically, the functional asymmetry in the right-lateralized enhanced envelope coding shown here presumably occurs at the cortical level (Poeppel, 2003; Luo and Poeppel, 2007), adding to the increasing evidence that auditory pathology results in changes in the central auditory system (Morita et al., 2003; Wienbruch et al., 2006; Wilding et al., 2012; Alain et al., 2014; Auerbach et al., 2014).

The present results reveal that the fidelity of cortical envelope coding was also enhanced in SNHL listeners compared with NH listeners. Fast-acting compression present in the basilar membrane of NH listeners distorts the shape of envelope coding (Stone and Moore, 2007). A reduction or loss of fast-acting compression in SNHL listeners may reduce the distortions introduced into the coding of the temporal envelope of sounds at the level of the basilar membrane, resulting in increased fidelity of envelope coding in SNHL listeners. Alternatively, the broadened auditory filters of SNHL listeners may reduce the influence of the inherent fluctuations present in noise maskers (Oxenham and Kreft, 2014; Stone and Moore, 2014), leading to the improved fidelity of envelope coding measured in SNHL listeners in the present study.

### Summary

Understanding speech in modulated noise is particularly difficult for hearing-impaired listeners with SNHL. We used MEG to investigate how modulated noise is represented in auditory cortex of NH and hearing-impaired listeners. Enhanced envelope coding was associated with a perceptual deficit: The amplitude of cortical envelope coding of modulated noise was linked with speech identification in the presence of modulated noise. Magnified cortical envelope coding may cause deficits in speech perception in modulated background noise because it disrupts the segregation of speech from a modulated background noise.
